# Recovery of severe COVID-19 complicated with cerebral infarction: Considerations on a case report

**DOI:** 10.1097/MD.0000000000033870

**Published:** 2023-05-26

**Authors:** Erli Mao, Zhaohui Qu, Juan Jin, Cui Yao, Wenjun Lyu, Feng Jiang, Hui Ding, Chuyan Wu

**Affiliations:** a Department of Rehabilitation Center, The First Affiliated Hospital of Nanjing Medical University, Nanjing, China; b Department of Critical Medicine, Wuhan Jinyintan Hospital, Wuhan, Hubei, China; c Department of Colorectal Center, The First Affiliated Hospital of Nanjing Medical University, Nanjing, China; d General Practice Department, The Geriatric Hospital of Nanjing Medical University, Nanjing, China; e Department of Neonatology, Obstetrics and Gynecology Hospital of Fudan University, Shanghai, China.

**Keywords:** COVID-19, tracheotomy, cerebral infarction, rehabilitation nursing

## Abstract

The standardization, individualization, and rationalization of intensive care and treatment for severe patients have improved. However, the combination of corona virus disease 2019 (COVID-19) and cerebral infarction presents new challenges beyond routine nursing care.

**Patient concerns and diagnoses:**

This paper examines the rehabilitation nursing of patients with both COVID-19 and cerebral infarction as an example. It is necessary to develop a nursing plan for COVID-19 patients and implement early rehabilitation nursing for cerebral infarction patients.

**Interventions:**

Timely rehabilitation nursing intervention is essential to enhance treatment outcomes and promote patient rehabilitation. After 20 days of rehabilitation nursing treatment, patients showed significant improvement in visual analogue scale score, drinking test, and upper and lower limb muscle strength.

**Outcomes:**

Treatment outcomes for complications, motor function, and daily activities also improved significantly.

**Lessons:**

Critical care and rehabilitation specialist care play a positive role in ensuring patient safety and improving their quality of life by adapting measures to local conditions and the timing of care.

## 1. Introduction

The standardization, individualization, and streamlining of intensive care and treatment for severe patients have improved, and it is crucial for minimizing complications and improving the quality of life of patients infected with severe acute respiratory syndrome coronavirus 2. However, the combination of corona virus disease 2019 (COVID-19) and cerebral infarction has introduced a new level of complexity beyond routine nursing care. In this article, a clinical case study is presented of a COVID-19 patient who was admitted to the Intensive Care Unit with a tracheotomy and cerebral infarction. The patient received intensive care and early recovery support during the early stages of the disease. Rehabilitation nursing experience is beneficial for aiding in the recovery of different functions for seriously ill patients. The following is a summary of the nursing methods and experience used in this case.

## 2. Case report

A 62-year-old male patient was transferred to Jinyintan Hospital on January 29th due to intermittent 8-day fever and 4-day asthma. A positive nucleic acid test confirmed a diagnosis of COVID-19. The patient had a 20-year history of hypertension but did not take medications regularly. Additionally, the patient had a history of coronary heart disease for 6 years and was taking oral lipid-lowering drugs.

1. As mask oxygenation failed to alleviate the patient’s symptoms, they received endotracheal intubation and mechanical ventilation. The results of the laboratory and facility inspections are presented in Table 1.
2. Progress in the course of disease is shown in Table 2.
3. The treatment of drugs is shown in Table 3.

**Table 1:** Results of the laboratory and facility inspections.

Date	Inspection item		Results
01–30	Blood routine	lymphocyte percentage	4.1%
		Absolute lymphocyte count	0.33 × 10^9^/L
		Ferritin	>200 0.00ng/mL
	IL-6	Prointerleukin 6 assay	100.95pg/mL
01–30	Coagulation	D-dimer assay	1.74ug/mL
02–04	Chest CT		Lobar consolidation in upper lobe of right lung and lower lobe of both lungs, bilateral pleurisy
03–08	Cranial CT		Suggesting cerebral infarction
03–04	Sputum culture		Klebsiella pneumoniae
	Blood culture		Acinetobacter baumannii
03–20	Biochemical	Albumin	28g/L
03–30	Chest X-ray		Extensive patchy, flocculent shadows with blurred edges were seen in each lung field of both lungs

IL-6 = interleukin 6, CT = computed tomography.

**Table 2:** Progress record.

Date	Progress of disease course
01–30	The patient was diagnosed as COVID-19 with a temperature of 38.5°C and positive nucleic acid test. The patient was given mechanical ventilation through endotracheal intubation because oxygen inhalation by mask could not improve.
02–04	Repeated high fever, the highest body temperature 38–39 degrees, elevated hemogram, inflammatory indicators, consider serious infection, use more than 2 antibiotics combined treatment.
02–19	Tracheotomy
03–04	Sputum culture suggested Klebsiella pneumoniae and blood culture suggested Acinetobacter baumannii.
03–08	Chest CT: Bilateral pulmonary infectious lesions, considering viral pneumonia, right upper lobe and 2 lower lobe lobar lung consolidation, bilateral pleurisy. Cranial CT: suggesting cerebral infarction, the patient had right hemiplegia. Low molecular weight heparin calcium 4200u anticoagulation therapy.
03–12	Fiberoptic bronchoscopy aspirates sputum.
03–15	Successfully weaned from the ventilator, and the tracheotomy cannula was given 6 L/min oxygen inhalation.
03–20	Recovery plasma therapy.
03–23	Ambiguous mind, depression, 6 L/min oxygen inhalation in tracheal cannula, 2-degree thick sputum, 3-grade muscle strength of left upper limb, 1-Grade left lower limb, 0-grade right upper limb, and 0-grade lower limb; indwelling gastric tube; indwelling urinary catheter. Vital signs T37.8, HR110 times/min, RR18 times/min, BP125/60 mm Hg, SPO2 98%.
03–30	Novel coronavirus nucleic acid test was negative for 3 consecutive times (all intervals of more than 24 h), and novel coronavirus antibody IgM and IgG were positive. Chest X-ray showed extensive patchy and flocculent shadows with blurred edges in each lung field of both lungs.
04–11	The Chinese Health Commission expert panel discussed and concluded that the patient had been cured of COVID-19.
From 03–23 to 04–11	At the same time of strengthening disinfection and isolation, nutritional support and psychological nursing were added. During the 20 days of intensive rehabilitation nursing, the patient was conscious and able to answer the questions correctly. The patient balanced in the sitting position at level 1 and drank water through the mouth 5mL/ time. The muscle strength of the left upper limb was level 5, the left lower limb was level 5, the right upper limb was level 2, and the right lower limb was level 2. ICF General Version [1] seven first score (62), ICF score (44).

COVID-19 = corona virus disease 2019, CT = computed tomography, T = temperature, HR = heart rate, RR = respiratory rate, BP = blood pressure,SPO2 = pulse oxygen saturation, IgM = immunoglobulin M, IgG = immunoglobulin G, ICF = international classification of functioning and disability.

**Table 3:** Medication table for patients.

Purpose of treatment	Drug name and dose	Route of administration	Treatment cycle
Antibacterial therapy	Cefoperazone tazobactam2g bid	Intravenous drip	01.29–02.04
	Linezolid0.6g q12h	Intravenous drip	02.04–02.08
	Polymyxin B 1 million U qd	Intravenous drip	03.01–03.21
	Steady Credible1g q12h	Intravenous drip	03.10–03.26
Resolving phlegm and relieving asthma	Ambroxol Injection 30 mg bid	Intravenous Bolus	01.29–04–11
Hypotension	Amlodipine Besylate Tablets 5 mg qd	Oral	01.29–04–11
Hypolipidemia	Alvastatin Calcium Tablets 20mg qd	Oral	01.29–04–11
Anticoagulant therapy	Low molecular weight heparin calcium 4200u qd	Subcutaneous injection	03.08–04.11
Enhanced immunity	Human immunoglobulin injection 20g qd	Intravenous drip	04.04–04.11

This case study has passed the clinical research project review of the Medical Ethics Committee of Wuhan Infectious Disease Hospital (Wuhan Jinyintan Hospital) (ethics number KY-2020-84.01). The detailed content of this case report has been obtained with the informed consent of the patient and his legal guardian.

## 3. Interventions

The treatment of COVID-19 primarily targets the lungs, and patients with cerebral infarction commonly experience decreased lung function. The combination of these conditions presents a significant challenge in terms of pulmonary rehabilitation for the patient.

### 3.1. Intestinal treatment: Abdominal distension (needed urgent treatment)

**Figure 1.**
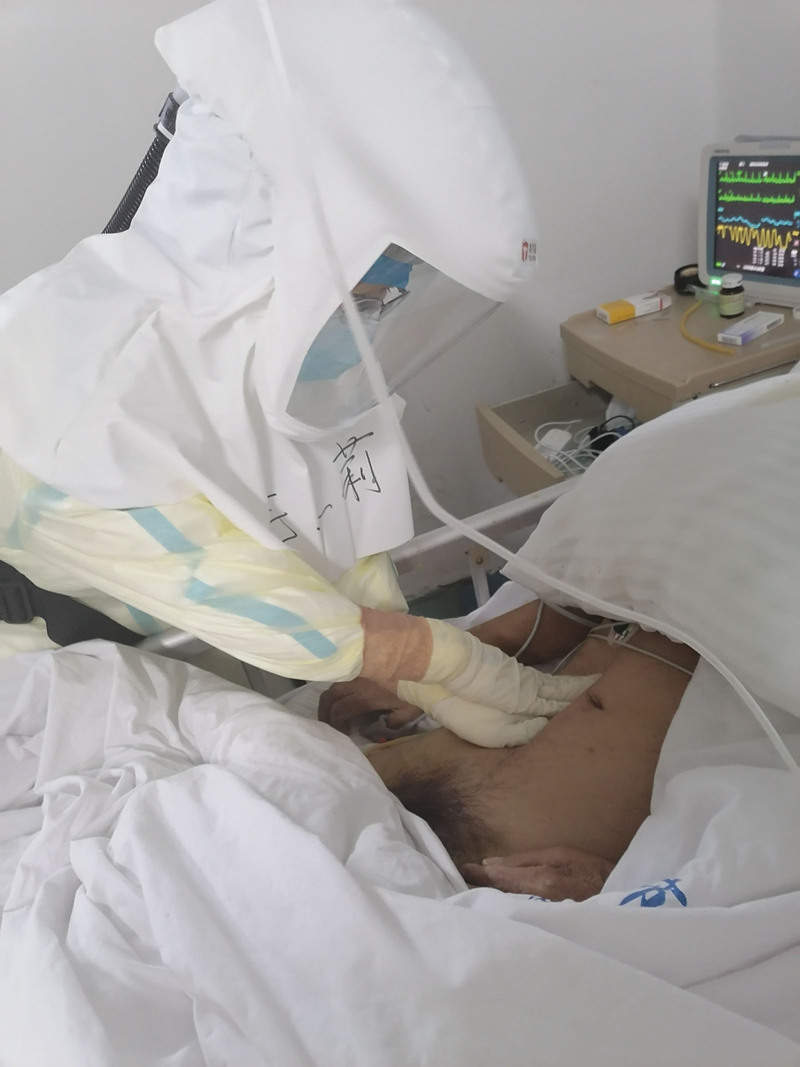
Intestinal management.

The patient’s heart rate is currently at 110 beats per minute, with a respiratory rate of 40 beats per minute and an peripheral capillary oxygen saturation level of 90% (while receiving 5L/minutes oxygen through an intratracheal tube). The patient is irritable and blood gas analysis indicates type I respiratory failure. Upon physical assessment by a rehabilitation specialist nurse, the patient’s abdomen appeared distended like a drum, and bowel sounds were absent. An abdominal X-ray was ordered and conducted near the patient’s bed, revealing incomplete intestinal obstruction. Rehabilitation nurses took charge of intestinal management after communicating with physicians (Fig. 1).

### 3.2. Methods

#### 3.2.1. Rectal stimulation

The patient was in a decubitus position on his left side, and the nurse put gloves on his right hand and gently massaged the anal margin with lubricant on the middle finger until slowly inserting the middle finger into the rectum between 4 to 5cm. The first nurse performed manual stool removal, removing about 30 bits of Bristol stool Type I1 and pressing them against the rectal wall at 3, 6, 9, and 12 o’clock. Each stimulation lasted 1 minute, with a 2-minute interval between them.

#### 3.2.2. Abdomen circular massage

The nurse performed a 10-minute massage on the ascending colon, transverse colon, descending colon, and sigmoid colon in a clockwise circular motion using 1 or both hands. This was followed by manual defecation, which was repeated twice, resulting in a total of 50 capsules. Afterward, the patient underwent 3 days of intestinal recovery preparation.

### 3.3. Effect evaluation

After the massage and manual defecation, the patient experienced relief from abdominal distension pain, relaxation, and restoration of gastrointestinal tract peristalsis. Three days later, the patient defecated spontaneously. The stool had a smooth appearance and was shaped like a sausage or banana, classified as fecal excretion type IV.

#### 3.3.1. Posture management^[1]^ (The most easily completed first condition for pulmonary rehabilitation)

The patient had been bedridden for nearly 45 days. The pulmonary rehabilitation posture management had to be paired with the hemiplegia after cerebral infarction posture management. The rehabilitation specialist nurse carried out a specific procedure.

### 3.4. Method

Throughout the day, the nurse raised the head of the bed by 30° to 45° and 45° to 60° and labeled it. The patient was gradually transitioned from a 30° to 45° bedhead elevated position to a 60° position after a brief adaptation duration of 1 to 2 hours. Before shaking the head of the bed, nurses should remember to shake the knee joint 10 to 15 degrees or place a small pillow under the knee.

We use antispasmodic positions for the patient in the evening and at night.

### 3.5. Effect evaluation

The patient’s traditional functional residual ability and airway resistance decreased after antigravity posture management.

#### 3.5.1. Management of artificial airway

When the patient removed the ventilator, the artificial airway transitioned to an open airway. To ensure safety, rehabilitation specialist nurses and critical care nurses must exercise caution in their self-protection and follow proper procedures for avoiding the spread of aerosols and droplets. Minimizing sputum suction stimulation while improving sputum suction performance is also crucial. Good communication with doctors is vital in this process.

### 3.6. Methods

1. To secure the tracheal tube, 1 finger was inserted into the fixation belt. In order to protect the skin around the collar, clean gauze was placed over the fixation belt. The tracheotomy opening was disinfected with 0.5 percent iodine and covered with sterile gauze twice daily for protection.
2. During sputum suction, a closed sputum suction pipe was used, with the other end of the Y-shaped pipe connected to an artificial nose, creating a closed loop to actively humidify the airway. The flushing side pipe was directly connected to the infusion set, which contained 250 mL of sterilized water for injection. This water was used to flush the sputum suction pipe after sputum aspiration.
3. Sputum aspiration: the method of shallow sputum aspiration^[2]^ with suction side intubation under the state of negative pressure is adopted. It is recommended to remove the secretions closest to the tracheotomy opening first, followed by inserting the sputum aspiration tube 1 to 2cm into the air-cut cannula. This technique minimizes the impact on intracranial pressure while allowing for quick clearance of airway secretions.

### 3.7. Effect evaluation

During the first week, the patient produced approximately 150 to 180 mL of sputum per day, which decreased to 50 to 100 mL per day in the subsequent weeks.

#### 3.7.1. Management of the tube balloon and training to improve speech function

During critical care, emphasis was placed on the role of tracheal cannula airbag control. The patient was unable to speak after the airbag was inflated for recovery purposes. The risks associated with this procedure were high, and after consulting with the doctor, the nurse needed to carefully monitor the patient’s oxygenation index. This approach was introduced through collaboration between critical care and recovery specialist nurses.

### 3.8. Methods

After the airbag was properly deflated, the nurse occluded the cannula with a finger to facilitate pronunciation.^[3]^ However, to avoid cross-infection and protect both patients and nursing staff, the use of artificial noses to occlude the cannula nozzle was attempted instead of using fingers. In this method, the cannula nozzle was partially blocked, allowing breathing airflow to be reintroduced into the larynx, enabling the patient to pronounce via the vocal cords. The patient was also taught to regulate breathing using the tube. Oxygen saturation of the patient’s pulse was monitored throughout the process.

### 3.9. Effect evaluation

After 1 week of airbag control and speech function instruction, the patient was able to speak 2 to 3 words consecutively. Two weeks later, he was able to connect with his daughter over the phone.

#### 3.9.1. Swallowing management

Following a complicated cerebral infarction, the patient developed dysphagia, which prevented them from eating orally. The development of aspiration pneumonia was largely attributed to the deterioration of oral self-purification and the reproduction of harmful bacteria in the oral cavity. The rehabilitation nursing specialist nurse will provide dysphagia functional training at this stage, after effective communication with the doctor. Meanwhile, the critical care nurse will be responsible for ensuring adequate oral care.^[4]^

### 3.10. Methods

#### 3.10.1. Strengthen oral care

Critical care nurses are responsible for providing patients with high-quality oral care. This includes brushing the patient’s teeth with a toothbrush and saline solution,^[5]^ 3 times a day.

#### 3.10.2. Modified water swallow test ^[6]^

The Modified water swallow test assessment was conducted to evaluate the patient’s swallowing function. The drinking time was measured at 5 seconds. The test volume was initially set at 1 mL, and deemed successful if the patient did not experience coughing or choking while drinking the water. Based on the results, an oral dosage of 5 mL was prescribed for the patient. The patient was also advised to perform regular oral exercises, including mouth agape, lips pursed, lips extended, and lips pursed again, with 1 series of 15 exercises to be done twice daily to improve swallowing function. Drink water plan^[7]^: Twice daily, the patient should consume clean water after performing thorough oral care. The total amount of water should be limited to 90 mL each time.

### 3.11. Effect evaluation

The patient was able to drink 90 mL of clean water independently from a water cup without experiencing coughing or choking.

#### 3.11.1. Pulmonary rehabilitation nursing

Breathing training^[8,9]^is the top priority for patients with pulmonary rehabilitation. The resolution of issues such as abdominal distension, posture management, speech therapy, and swallowing management has laid the foundation for pulmonary rehabilitation. This process should be carried out step-by-step under the supervision of vital nurses, with specialized recovery nurses overseeing the procedure after successful consultation with the doctor.

### 3.12. Methods

1. Patients were aided in performing abdominal (diaphragmatic) breathing exercises for 3 to 5 sets after a 30 to s45° increase in respiratory training. The rest period was gradually increased to 5 to 20 minutes.
2. Thoracic compliance.

To facilitate thoracic movement during breathing, the patient’s left and right sides should be touched with both hands. When the patient completes inhalation, the hands should be used to push up and inward to artificially lift the diaphragm and improve thoracic compliance.

1. Patient acquired weakness.^[10]^ If the patient is unable to cooperate with effective cough training, the nurse must assist them in adopting forced exhalation techniques, which involves 1 or 2 “hushs.” This technique aids in moving peripheral secretions to the upper airway and bronchus with expiratory airflow until they reach the airway’s opening. Respiratory training should be introduced gradually, and changes in the patient’s heart rate and Peripheral capillary oxygen saturation should be monitored. The patient’s Rating of Perceived Exertion score should range from 11 to 13, indicating no apparent fatigue during training.

### 3.13. Effect evaluation

During breathing preparation, the patient exhibited strong breathing control and cooperated well. The patient spontaneously discharged sputum to the tracheotomy’s open end, and there were no signs of exhaustion (Rating of Perceived Exertion score 11 to 13 points).

## 4. Outcomes

After receiving 20 days of rehabilitation nursing care, the patient’s visual analogue scale score, drinking test, and upper and lower limb muscle strength all exhibited significant improvement. The patient also experienced better outcomes in the treatment of complications, motor control, and activities of daily living. Additionally, speech therapy was instrumental in helping the patient regain language abilities.

## 5. Discussion

Despite transitioning from the acute to the rehabilitation process, the patient’s condition had not yet stabilized. Due to the isolation wards, the current form of incorporating rehabilitation nursing technologies for patients with severe COVID-19 disease is limited. To address this issue, a principle of “enhanced clinical and rehabilitation management” is being followed. Rehabilitation specialist nurses are working closely with physicians to provide early rehabilitation treatment and care to serious patients.

Under the strict supervision of critical care nurses, this patient was given regular early and intensive rehabilitation treatment by rehabilitation specialist nurses.^[11]^ The patient’s medical condition was still unstable when transitioning from the acute to the rehabilitation phase. Due to the principle of “enhanced clinical and rehabilitation management,” incorporating current rehabilitation nursing technologies for severe COVID-19 patients within isolation wards is limited. However, after consulting with physicians, rehabilitation specialist nurses can provide early rehabilitation treatment and care to serious patients.

Providing rehabilitation nursing poses certain challenges as it requires prolonged close contact with patients during language function, pulmonary, swallowing, and intestinal rehabilitation training. Nurses must wear 3 layers of protection, including heavy protective equipment, positive pressure headgear, and heavy protective clothing, which can have an impact on their physical health. This is a new challenge that rehabilitation nurses must overcome.

To sum up, personalized care and treatment are crucial for patients with severe COVID-19. Collaborative efforts between critical care and rehabilitation specialists are essential to ensure patient safety and enhance their quality of life by adapting interventions to the specific needs and context of each patient.

## Author contributions

**Conceptualization:** Feng Jiang, Hui Ding.

**Data curation:** Erli Mao, Zhaohui Qu, Juan Jin, Cui Yao, Feng Jiang.

**Formal analysis:** Erli Mao, Cui Yao.

**Investigation:** Juan Jin, Cui Yao, Wenjun lyu, Hui Ding.

**Methodology:** Chuyan Wu.

**Writing – original draft:** Hui Ding, Chuyan Wu.

**Writing – review & editing:** Feng Jiang, Chuyan Wu.
